# Research on High Performance Methane Gas Concentration Sensor Based on Pyramid Beam Splitter Matrix

**DOI:** 10.3390/s24020602

**Published:** 2024-01-17

**Authors:** Boqiang Wang, Xuezeng Zhao, Yiyong Zhang, Zhuogang Wang

**Affiliations:** 1 School of Mechatonics Engineering, Harbin Institute of Technology, Harbin 150006, China; zhaoxz@hit.edu.cn (X.Z.); 17601527196@163.com (Y.Z.); 2 703 Research Institute, China State Shipbuilding Corporation Limited, Harbin 150010, China; 15124588116@163.com

**Keywords:** methane gas concentration detection, redundant sensor, pyramidal beam splitter matrix, signal superposition technology, chirp z-transform

## Abstract

Methane gas concentration detection faces the challenges of increasing accuracy and sensitivity, as well as high reliability in harsh environments. The special design of the optical path structure of the sensitive element provides an opportunity to improve methane gas concentration detection. In this study, the optical path structure of the sensitive element was newly designed based on the Pyramidal beam splitter matrix. The infrared light source was modulated by multi-frequency point-signal superimposed modulation technology. At the same time, concentration detection results and confidence levels were calculated using the four-channel methane gas concentration detection algorithm based on spectral refinement. Through the experiment, it was found that the sensor enables the full-range measurement of CH4; at the lower explosive limit (LEL, CH4 LEL of 5%), the reliability level is 0.01 parts-per-million (PPM), and the limit of detection is 0.5 ppm. The sensor is still capable of achieving PPM-level detections under extreme conditions in which the sensor’s optical window is covered by two-thirds and humidity is 85% or dust concentration is 100 mg/m^3^. Those improve the sensitivity, robustness, reliability, and accuracy of the sensor.

## 1. Introduction

Fires and even explosions caused by methane gas leaks are threats to safety production in the petrochemical field, which accounts for about 50% of all major petrochemical accidents in China [1,2,3]. Although methane gas sensors are prevalent in the workplace and have alarmed considerable staff for emergency response, about 40% of flammable and explosive accidents in petrochemical production sites were caused by no or failed alarms [4,5]. On the other hand, there were over 300,000 alarms in China responded to by fire departments in 2022 due to sensor malfunctions, leading to disruption to normal production, the spending of social resources, and decreasing public confidence [6,7]. Prompt and reliable methane gas leak detection is critical for rescuing life and avoiding flammable and explosive damage.

Methane gas concentration detection technology faces the challenges of improving detection limits, false alarm resistance, and adaptation under extreme environments. Increasing the optical path length is the key instrument for improving sensitivity, involving techniques such as reflective optics [8] and compact pentahedron reflector structures [9] that increase the light path length through the reflection process. In addition to that, detection limits can be improved by increasing the efficiency of the sensitive element, for instance, by plating zirconate titanate (PZT) film on the surface of the sensitive element [10] and etching 3D patterns on the Monocrystalline lithium tantalate film [11]. Furthermore, improving the infrared light source efficiency is another way to improve detection limits, for example, by installing high-refractive-index long period gratings (LPFG) in front of an infrared light source [12] and improving the infrared light source structure based on the Fabry-Borot (FPG) structure [13]. All the methods above can achieve sub-ppm-level detection. However, at the very beginning of the leak, the concentration of methane gases is below the PPM level; at the same time, the calculation results need to be evaluated at extremely low concentrations. In more serious cases, the reliability of the sensor will be affected when the window of the sensor is attached by contaminants or the infrared detection channel is aging or malfunctioning.

In this study, a four-channel infrared sensitive element with a new optical structure was designed to improve sensor accuracy, sensitivity, and reliability. The pyramidal beam splitter matrix was used as an optical reflection structure inside the sensitive element. On the one hand, the sensitivity of the sensor was improved by increasing the optical path length. On the other hand, the complex optical reflection path enabled the four-signal detection channel to receive a uniform infrared light signal and effectively reduced the impact of contaminants (such as condensation fog, dust, etc.) attached to the optical window on the performance of the sensor, thus raising the reliability of the sensor. The concentration calculation results and the confidence level were calculated via the results of the four infrared signal detections that were calculated by the four-channel methane gas concentration redundancy algorithm based on the linear frequency modulation chirp z-transform (CZT). The designed methane gas concentration sensors were tested for detection performance, accuracy and sensitivity, and reliability through the methane gas concentration calibration experiment, the methane gas limit detection experiment, and the anti-interference capability simulation experiment, respectively.

## 2. Design of Sensitive Element and Measurement Circuit

### 2.1. Sensitive Element Design

**Figure 1:**
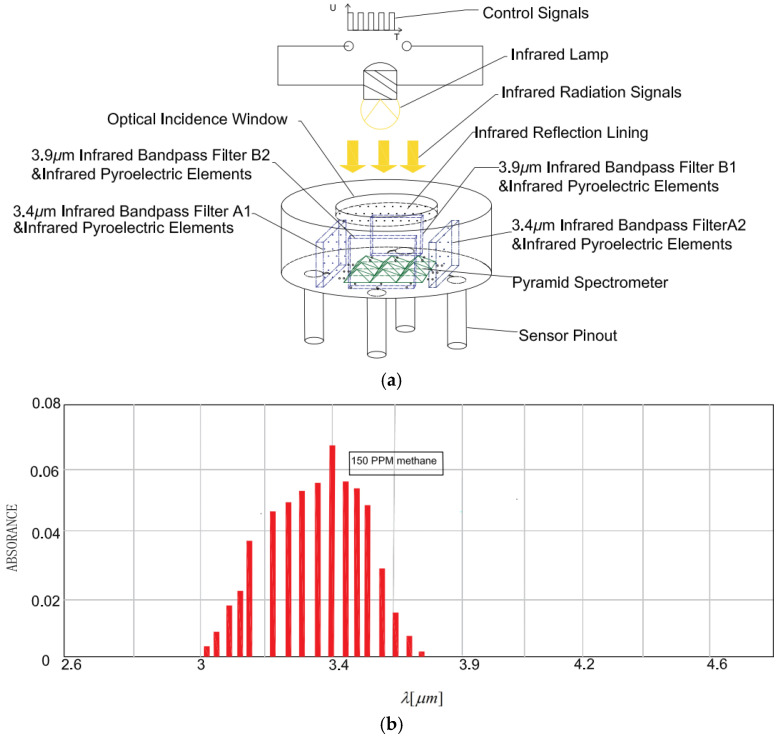
Element structure and methane absorption spectrum; (**a**) Schematic diagram of the structure of the sensitive element; (**b**) Methane absorption spectrum.

In order to increase the optical length and mix infrared light well, as shown in Figure 1, a pyramid beam splitter matrix is added in the center of the sensitive element. At the same time, the combination of four infrared bandpass filters and infrared pyroelectric elements is placed around the pyramidal beam splitter matrix. An optical incidence window is arranged at the top of the sensitive element, with an infrared reflective lining on the inside. The infrared reflective lining has high pass-through to infrared light signals from the entrance to the inside of the sensitive element, and it has full-spectrum high reflectivity for infrared light signals reflected by the pyramidal spectral matrix and infrared bandpass filter.

As shown in Figure 1b, the methane gas only absorbs infrared light signals near the center wavelength of 3.4 μm and does not absorb infrared light signals near the center wavelength of 3.91 μm. Therefore, infrared bandpass filters with a center wavelength of 3.4 μm and a bandwidth of 0.2 μm are mounted in front of the two sensitive elements of the detection channel to detect the methane gas concentration. Infrared bandpass filters with a center wavelength of 3.91 μm and a bandwidth of 0.2 μm are installed in front of the two sensitive elements of the reference channel to provide a reference for the detection channel.

**Figure 2:**
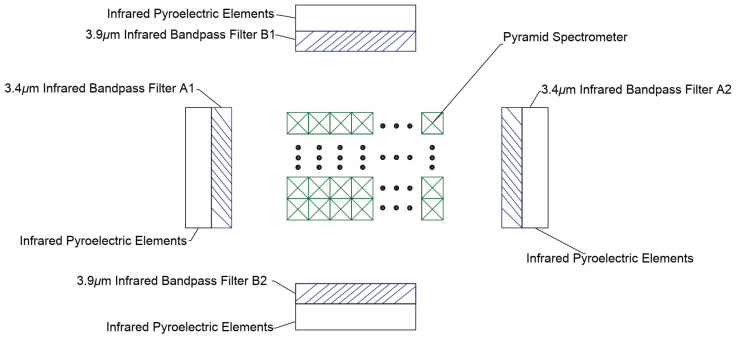
Top view of the inside of a sensitive element.

The top view of the inside of the sensitive element is shown in Figure 2. An infrared pyroelectric sensitive element is arranged behind each group of infrared bandpass filters (A1, A2, B1, B2). A1 and A2 only pass through the infrared light signal with a center wavelength of 3.4 μm and a bandwidth of 0.2 μm for the detection of methane gas. B1 and B2 only pass through the infrared light signal with a center wavelength of 3.91 μm and a bandwidth of 0.2 μm to provide a reference for the detection waveform. A1, A2, B1, and B2 have full-spectrum high reflectivity for infrared light signals outside the allowed passage wavelength.

**Figure 3:**
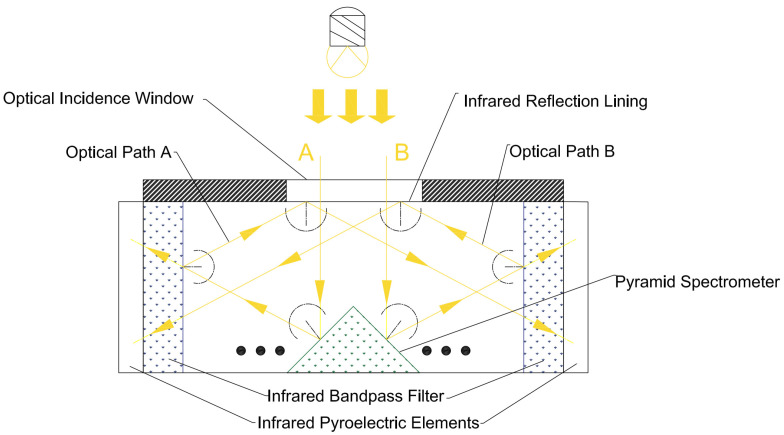
Schematic diagram of the optical path reflected by the pyramid beam splitter matrix.

As shown in Figure 3, infrared light A and B, to be emitted by the infrared light source, are shot inside the sensitive element through the signal inlet on it. The infrared light is reflected toward the infrared bandpass filter when it hits the pyramidal beam splitter. Only the infrared light, within the bandpass wavelength range, can pass through it and shoot to the infrared pyroelectric element behind it. The rest of the infrared light, out of the bandpass wavelength range, will all be reflected into the infrared reflection lining, and a secondary reflection—all the infrared light will be reflected, due to its highly reflective full spectrum—occurs here. The secondary reflected infrared light will be reflected to the bandpass filter in the opposite direction and the above optical reflection process will be repeated.

In Figure 3, only the reflection process for two beams of light (A and B) is shown between the two infrared bandpass filters and infrared reflection lining. Actually, it is a complex reflection process wherein a beam of light is reflected between the pyramid beam splitter matrix, four infrared bandpass filters (A1, A2, B1, and B2), and the infrared reflection lining. This process can increase the optical path length and provide all infrared light signals in the respective wavelength range to four infrared bandpass filters (A1, A2, B1, and B2).

As mentioned above, this design for the sensitive element can improve the reliability and accuracy of the methane gas concentration sensor.

On the one hand, we discuss the impact of improving the accuracy of the sensor for the detection of methane gas concentration. Absorption properties follow the Beer–Lambert law [14,15]:

(1) $$I=I_{0}e^{-\alpha CL}$$

Here, $I_{0}$ is the intensity of the infrared light incident, I is the intensity of the infrared light transmitted through the gas, α is the absorption coefficient of the gas, L is the optical path length, and C is the concentration of the gas. Apparently, increasing the optical path length can improve the absorption of infrared light by the methane gas, when the concentration of the methane gas is certain, and improve the accuracy of the sensitive element to detect methane gas concentration. The complex optical reflection process in the internal optical path structure of the sensitive element can increase the optical path length so that the infrared light signal can be fully absorbed by the methane gas. The electrical signal difference can be increased between the detection channel and the reference channel, thus increasing the accuracy of the sensor for the detection of methane gas concentration.

On the other hand, we discuss the impact of improving the reliability of the sensor. The condensation fog can be formed on the optical incidence window of the sensitive element due to the fact that the sensor is affected by environmental temperature factors (such as the temperature difference between day and night, the system between start and stop, etc.). And, dust particles are inevitably attached to the optical incidence window of the sensitive element on account of the fact that the sensor inlet and outlet are open paths. All of these conditions can lead to the varying reduction in infrared signals that are received by each of the infrared signal detection channels so that the detection malfunction occurs. The complex optical reflection process inside the sensitive element enables each of the infrared signal detection channels to receive a uniform signal. Even if the incidence window is attached to contaminants—it causes the non-uniform incidence of infrared light signal into the sensitive element—each of the infrared signal detection channels can receive the uniform signal, too. It thereby ensures that the sensor can effectively detect the concentration of methane gases even under unfavorable conditions, thus increasing the reliability of the sensor.

### 2.2. Measurement Circuit Design

**Figure 4:**
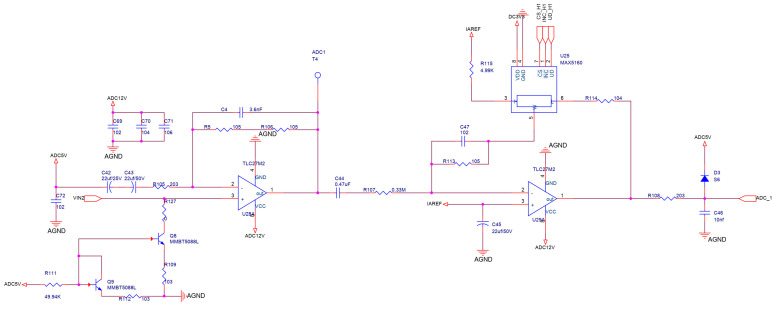
Schematic diagram of the measurement circuit.

The circuits of measuring signals from four detection channels of the sensitive elements are completely identical. This circuit diagram is shown in Figure 4. Therefore, only one of the detection circuits is used as an example. The circuit consists of two operational amplifiers (op. amps.) U28A and U29A, two transistors Q8 and Q9, and a digital potentiometer U25. The very-low-amplitude raw output at the sensitive element is amplified through the two-stage amplifier circuit that is constituted by U28A and U29A. And, these two transistors, Q8 and Q9, behave as a temperature compensation circuit that can suppress the temperature drift of the output signal from the sensitive component. The analog signal is precisely regulated by the digital potentiometer U25. Finally, the processed analog signal is passed to the analog-to-digital converters (ADCs).

## 3. Methane Gas Concentration Detection Algorithm Based on CZT Principle

### 3.1. CZT Algorithm Principle

The CZT algorithm is often used for spectrum refinement in the characteristic bands of the signal, and it has the advantages of flexible refinement scale and high accuracy.

The specific calculation process can be expressed as follows. Suppose signal sequences of limited length x(n) are spectrally refined in the frequency band with the origin frequency $f_{0}$ , end frequency $f_{L}$ , and bandwidth length M ; $M=f_{0}+f_{L}$ . This refinement is accomplished through CZT transformation [16,17,18,19,20,21].

(2) $$X(Z_{r})=CZT[x(n)]=\sum_{n=0}^{M-1}x(n)A_{0}^{-n}e^{-j\theta_{0}n}w_{0}^{-nr}e^{-j\varphi_{0}nr}$$

Here, $\theta_{0}$ is the initial amplitude angle, $\varphi_{0}$ represents equally spaced increments on a unit circle angle, $A_{0}$ is the length of the vector radius at the starting sampling point, $w_{0}$ is the elongation of the Z-plane helix, j is the imaginary unit, and the superscript r denotes the serial number sample on the unit circle, when $A_{0}$ and $w_{0}$ are equal to 1 at the same time. We can derive Equation (3) from Equation (2).

(3) $$X(r)=\sum_{n=0}^{M-1}x(n)\exp[-j\left(\theta_{0}+\varphi_{0}r\right)n]$$

Here, $\theta_{0}$ is equal to $2\pi f_{0}/f_{s}$ , $\varphi_{0}$ is equal to $2\pi f_{L}/(Mf_{s})$ and $f_{s}$ is the sampling frequency of the signal. Therefore, the frequency resolution of this band ∆f after refinement of the analysis is equal to $f_{L}/M$ .

### 3.2. Four-Channel Methane Gas Concentration Redundancy Calculation Model

**Figure 5:**
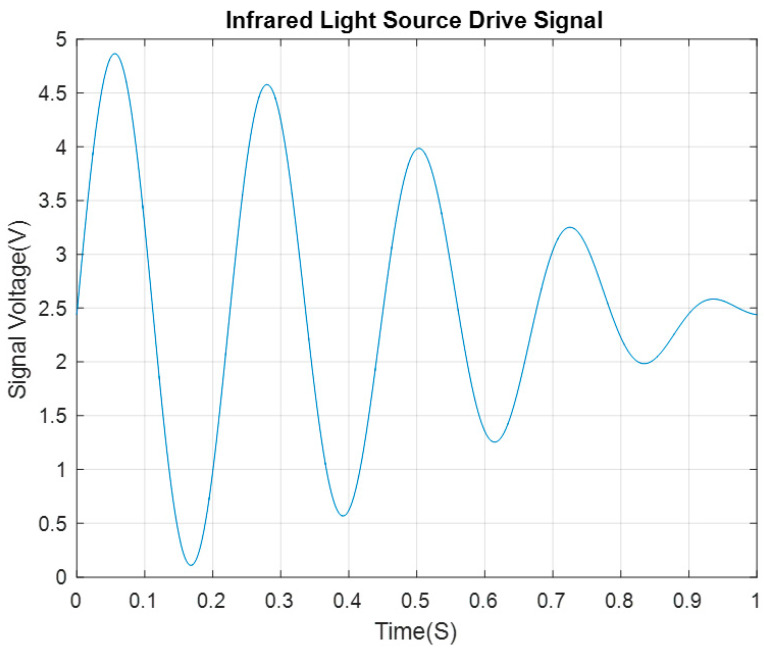
Infrared light source drive voltage signal timing diagram.

As shown in Figure 5, the drive signal of the infrared light is driven by the modulated waveform that is superimposed by the sinusoidal signal with a frequency of 4.0–5.0 Hz and an interval of 0.1 Hz.

In Figure 4, the characteristic frequency of the signal output from the secondary amplifier circuit is between 4 and 5 Hz, with an interval of 0.1 Hz. Consequently, this band (between 4 and 5 Hz) is the characteristic frequency band of the signal, and has the frequency refinement scale of 0.1 Hz. We refine the spectrum for the characteristic band using the CZT algorithm.

Suppose that the modulus sums of the four secondary amplified signals calculated through the CZT algorithm are $M_{1}$ , $M_{2}$ , $R_{1}$ , and $R_{2}$ on a minimum resolution of 0.1 Hz in the signal characteristic frequency band between 4 and 5 Hz. Here, $M_{1}$ and $M_{2}$ are the mode sums of the infrared pyroelectric element 3.4 μm band channel 1 and channel 2, respectively and $R_{1}$ and $R_{2}$ are the modulus sums of the infrared pyroelectric element 3.91 μm band channel 1 and channel 2, respectively. Then, the expression can be written as follows:

(4) $$\{\begin{array}{c} M_{1}=\sum_{Z_{r}=4.0}^{5.0}X(Z_{r})=\sum_{Z_{r}=4.0}^{5.0}\sum_{n=0}^{M-1}x(ADC_{1})\exp[-j(\frac{2\pi f_{0}}{f_{s}}+\frac{2\pi f_{L}}{Mf_{s}}r)n]\\ M_{2}=\sum_{Z_{r}=4.0}^{5.0}X(Z_{r})=\sum_{Z_{r}=4.0}^{5.0}\sum_{n=0}^{M-1}x(ADC_{2})\exp[-j(\frac{2\pi f_{0}}{f_{s}}+\frac{2\pi f_{L}}{Mf_{s}}r)n]\\ R_{1}=\sum_{Z_{r}=4.0}^{5.0}X(Z_{r})=\sum_{Z_{r}=4.0}^{5.0}\sum_{n=0}^{M-1}x(ADC_{3})\exp[-j(\frac{2\pi f_{0}}{f_{s}}+\frac{2\pi f_{L}}{Mf_{s}}r)n]\\ R_{2}=\sum_{Z_{r}=4.0}^{5.0}X(Z_{r})=\sum_{Z_{r}=4.0}^{5.0}\sum_{n=0}^{M-1}x(ADC_{4})\exp[-j(\frac{2\pi f_{0}}{f_{s}}+\frac{2\pi f_{L}}{Mf_{s}}r)n] \end{array}$$

Here, $ADC_{1}$ and $ADC_{2}$ are the voltages obtained from the two concentration detection circuits and $ADC_{3}$ and $ADC_{4}$ are the voltages obtained from the two reference channel circuits.

As such, from the four ratios of the modulus sums, two infrared pyroelectric 3.4 μm bands (methane detection bands) are compared to two 3.91 μm bands (reference bands), which are calculated by the CZT algorithm in the characteristic frequency band and can be represented as follows:

(5) $$\{\begin{array}{c} Q_{1}=\frac{M_{1}}{R_{1}}\\ Q_{2}=\frac{M_{1}}{R_{2}}\\ Q_{3}=\frac{M_{2}}{R_{1}}\\ Q_{4}=\frac{M_{2}}{R_{2}} \end{array}$$

Here, $Q_{1}$ , $Q_{2}$ , $Q_{3}$ , and $Q_{4}$ are the ratios of modulus between two detection channels and two reference channels.

Then, methane gas concentrations from four redundant combinations can be calculated as follows:

(6) $$\{\begin{array}{c} C_{1}=1-\frac{M_{1}}{R_{1}}\\ C_{2}=1-\frac{M_{1}}{R_{2}}\\ C_{3}=1-\frac{M_{2}}{R_{1}}\\ C_{4}=1-\frac{M_{2}}{R_{2}} \end{array}$$

Here, $C_{1}$ , $C_{2}$ , $C_{3}$ , and $C_{4}$ are the methane gas concentration from four redundant combinations.

Ultimately, the concentration of the methane gas to be detected can be calculated as follows:

(7) $$COL=\bar{Q}=\frac{C_{1}+C_{2}+C_{3}+C_{4}}{4}$$

Here, COL is the result of the methane gas concentration to be detected, and $\bar{Q}$ is the average of the concentrations of the four redundant combinations.

The trusted accuracy of the concentration can be evaluated via the concentration variance $S_{COL}^{2}$ . The trusted accuracy of the sensor is at the PPM level when $S_{COL}^{2}$ is equal to 0.0000001.

(8) $$S_{COL}^{2}=\frac{{\left(\bar{Q}-C_{1}\right)}^{2}+{\left(\bar{Q}-C_{2}\right)}^{2}+{\left(\bar{Q}-C_{3}\right)}^{2}+{\left(\bar{Q}-C_{4}\right)}^{2}}{4}$$

## 4. Experiments and Results

### 4.1. Methane Gas Concentration Calibration Experiments and Results

**Figure 6:**
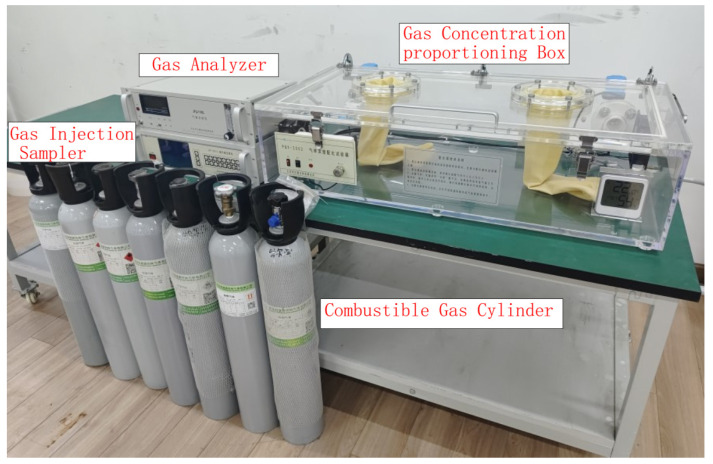
Methane gas concentration proportioning device diagram.

Methane gas from 0% to 90% LEL was produced by a proportioning device of methane gas concentration, as shown in Figure 6, and was used for the methane gas concentration calibration experiment involving the sensor. Among them, the methane gas with a concentration of 0% LEL was prepared by filling the methane gas concentration proportioning device with high-purity air. The proportioning accuracy of this device is 0.0001 PPM.

**Figure 7:**
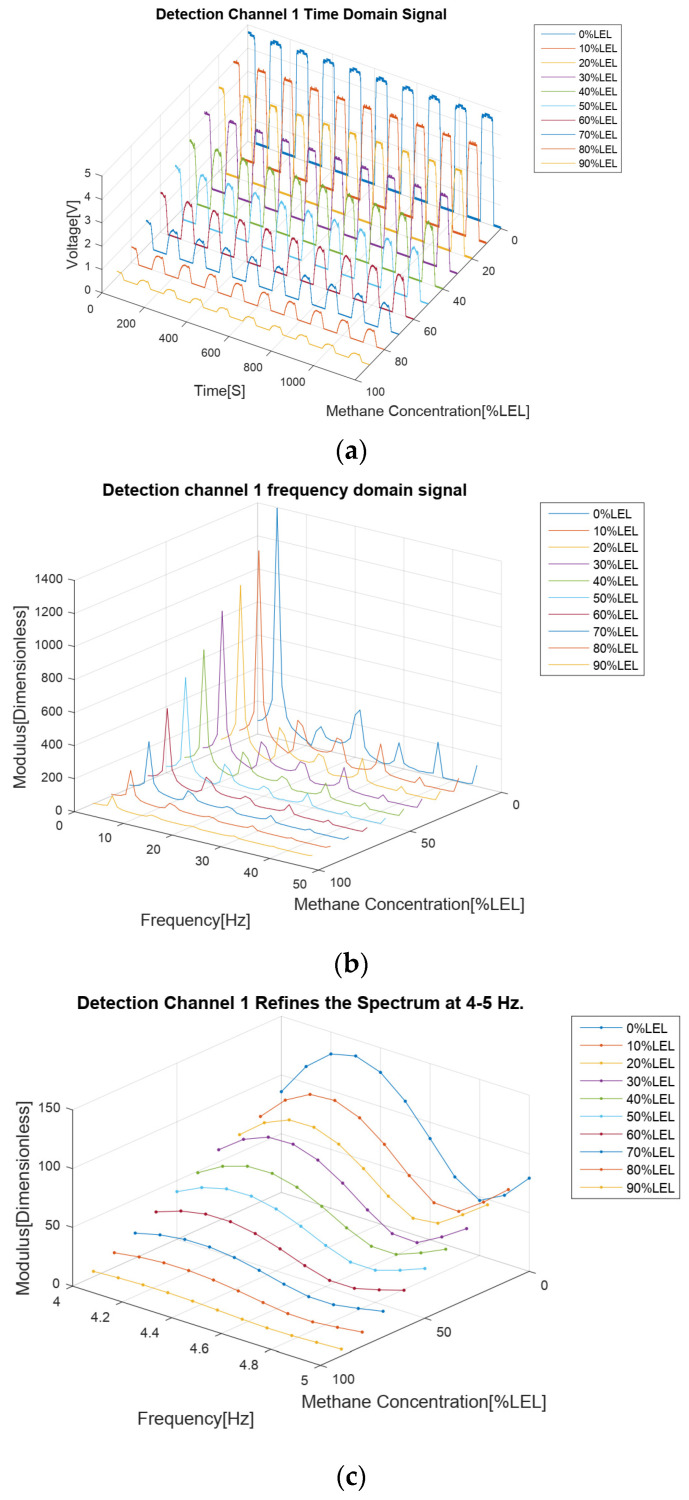
Methane gas detection channel 1 calibration test results for methane gas at 0–90% LEL concentration; (**a**) Time domain results; (**b**) Spectrum distribution; (**c**) 4–5 Hz frequency domain refinement results.

**Figure 8:**
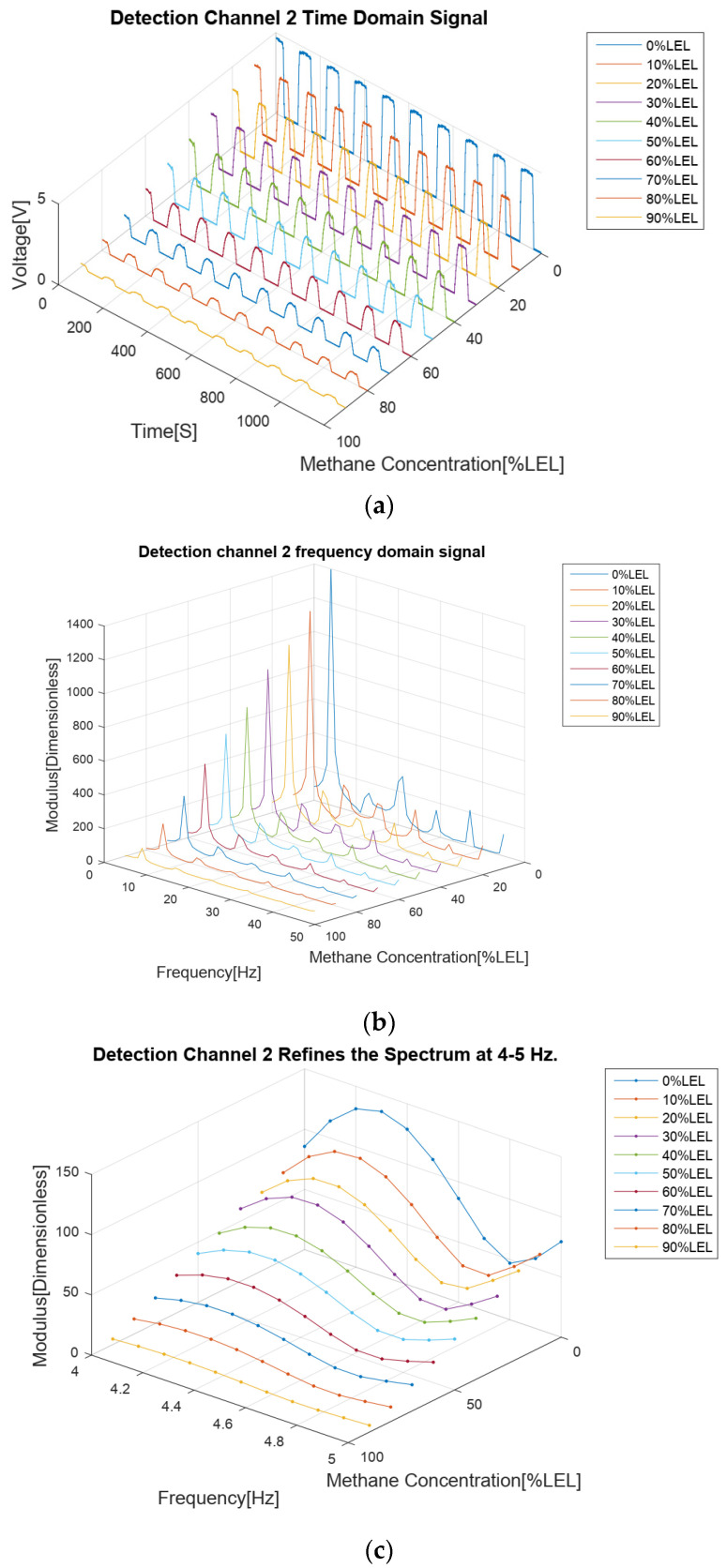
Methane gas detection channel 2 calibration test results for methane gas at 0–90% LEL concentration; (**a**) Time domain results; (**b**) Spectrum distribution; (**c**) 4–5 Hz frequency domain refinement results.

**Figure 9:**
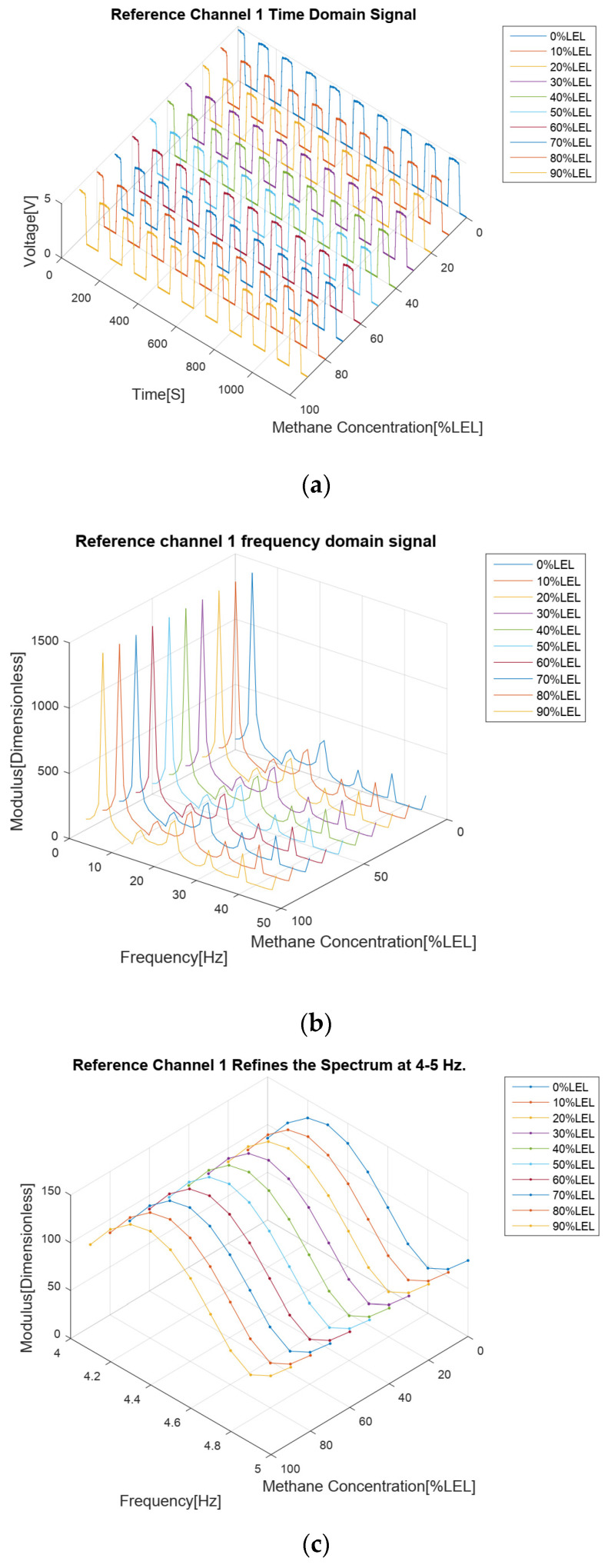
Methane gas reference channel 1 calibration test results for methane gas at 0–90% LEL concentration; (**a**) Time domain results; (**b**) Spectrum distribution; (**c**) 4–5 Hz frequency domain refinement results.

**Figure 10:**
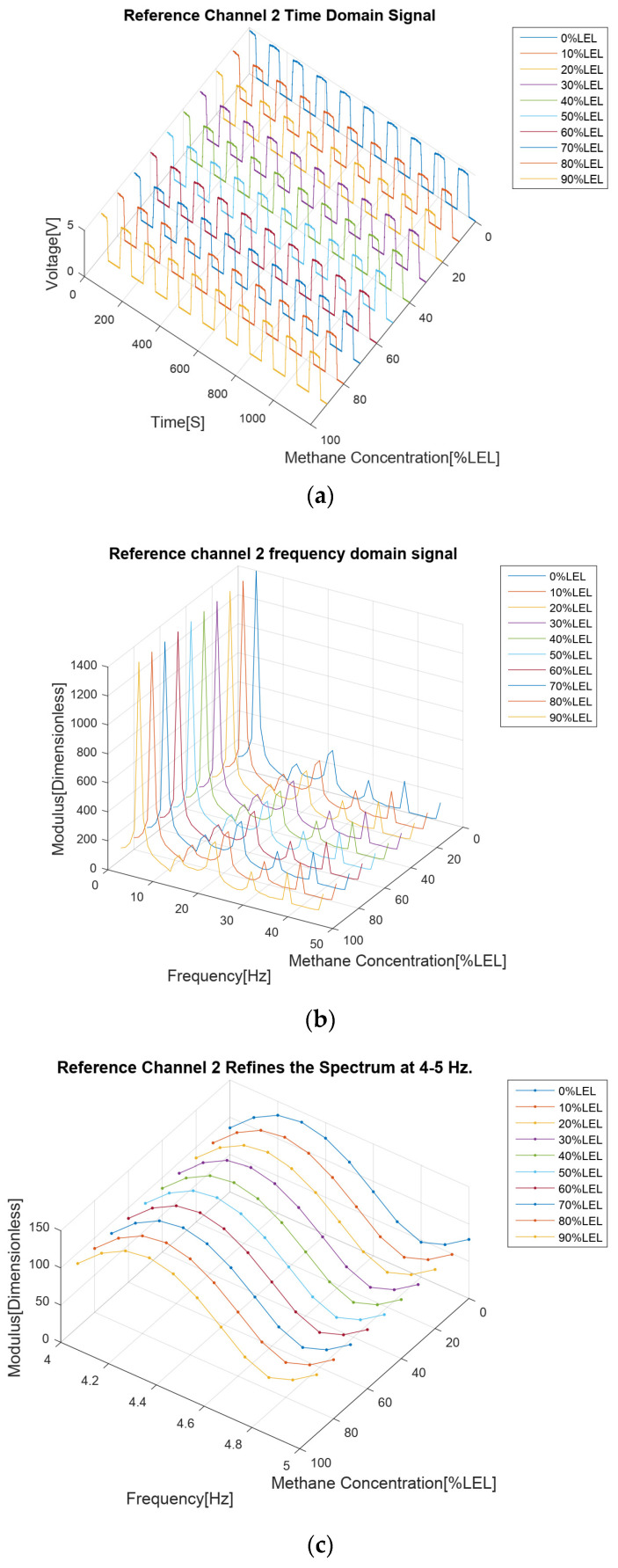
Methane gas reference channel 2 calibration test results for methane gas at 0–90% LEL concentration; (**a**) Time domain results; (**b**) Spectrum distribution; (**c**) 4–5 Hz frequency domain refinement results.

The results of the methane gas concentration calibration experiment are analyzed as follows. First of all, from the four time domain signal figures (Figure 7a, Figure 8a, Figure 9a and Figure 10a), we can find that the signal strengths of detection channels 1 and 2 decreased with the increase in the concentration of methane gas (Figure 7a and Figure 8a), and those of the two reference channels remained the same all the time (Figure 9a and Figure 10a).

In the follow-up phase of the spectrum analysis of the data for four channels, it can be found that spectrum peaks were concentrated between 4 and 5 Hz (Figure 7b, Figure 8b, Figure 9b and Figure 10b), indicating that the characteristic frequency band was in this band. Additionally, the spectrum peaks of detection channels 1 and 2 decreased with the increase in the concentration of methane gas (Figure 7b and Figure 8b), and those of the two reference channels remained the same all the time (Figure 9b and Figure 10b). The same conclusion was obtained above when the four channels were further analyzed in detail on the characteristic band (Figure 7c, Figure 8c, Figure 9c and Figure 10c).

For the final stage of the calculation of detection results for methane gas from 0% to 100% LEL concentration, it can be seen that the results of each concentration of methane gas were accurately calculated using the methane gas concentration detection algorithm based on the CZT principle (Table 1). The maximum value of the concentration variance $S_{COL}^{2}$ of the detection result was 0.014 PPM, and the minimum one was 0.0015 PPM, indicating that the trusted accuracy of the concentration detection result can reach a 0.014 PPM level in the most adverse condition.

### 4.2. Methane Gas Concentration Limit Detection Experiments and Results

**Figure 11:**
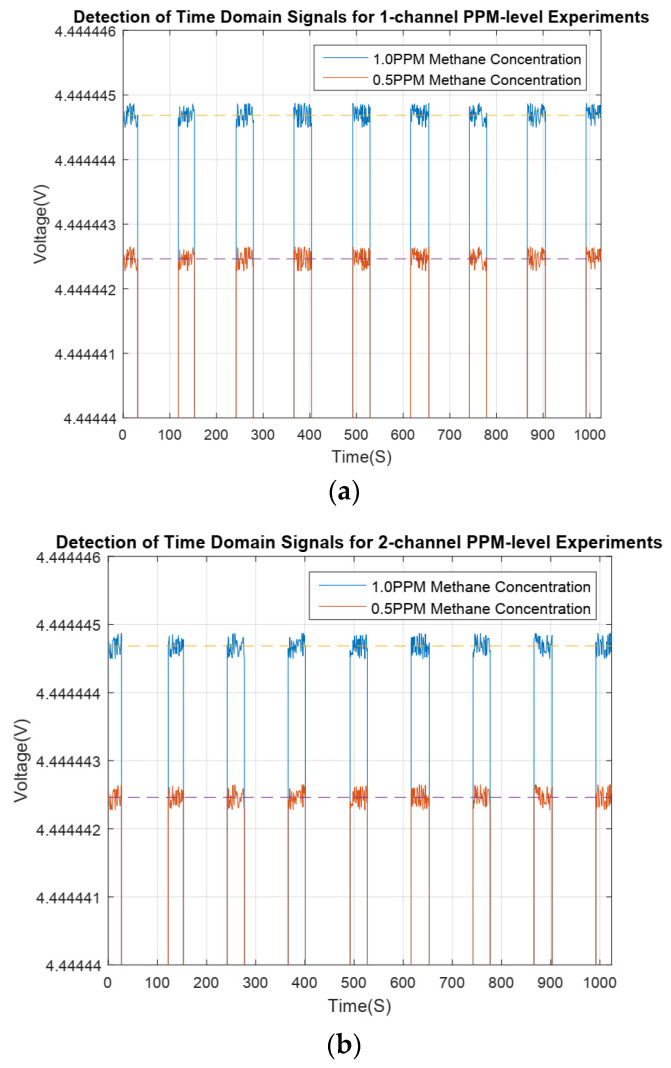
The time domain signals of the methane gas detection channels 1 and 2 at 0.5 PPM methane concentration; (**a**) Detection channel 1—time domain signal; (**b**) Detection channel 2—time domain signal.

Methane gas of the 0.5 PPM concentration was produced by a proportioning device of methane gas concentration and was used for the concentration limit detection experiment involving the sensor. See Figure 11.

As shown in Table 2, the result of the concentration, which was calculated using the methane gas concentration detection algorithm based on the CZT principle, was 0.5028 PPM, and the concentration variance $S_{COL}^{2}$ was 0.015 PPM. This indicates that the trusted accuracy of the concentration detection result is 0.01 PPM at least. Therefore, the concentration detection accuracy of the sensor can attain 0.01 PPM.

### 4.3. Anti-Interference Capability Simulation Experiments and Results

**Figure 12:**
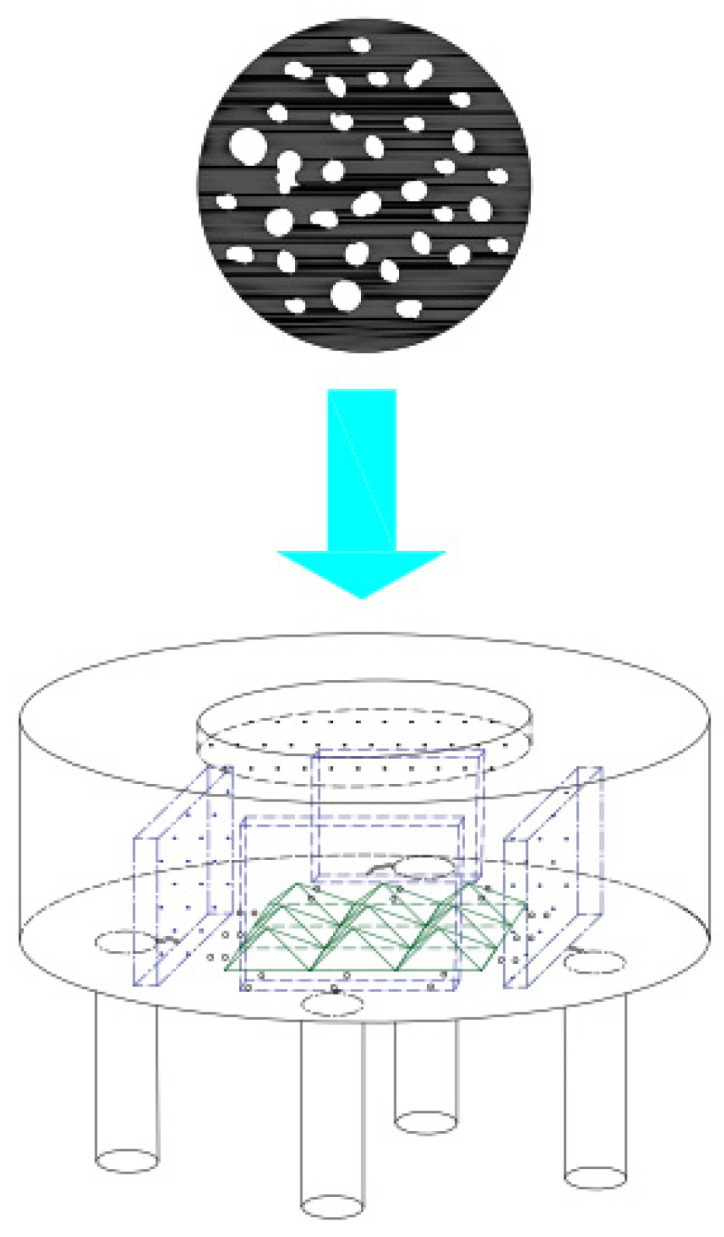
Installation diagram of the shading film.

The option incident window of the sensitive element was taped with the designed shading film so that it was simulated that this window was attached with contaminants (such as condensation fog and dust particles). The material of the shading film was a designed black polycondensation resin polarizer film and was randomly opened with several small holes that were two-thirds of the total area. In this way, one-third of the infrared light was incident of the sensitive element and another two-thirds of the infrared light were blocked from the sensitive element by the black overshadow film when the sensitive element was injected by the infrared light. See Figure 12.

Methane gas of the 30% LEL and 70% LEL concentrations was produced by a proportioning device of methane gas concentration and was used for the simulation experiment of anti-interference ability involving the sensor after being taped with the black shading film.

The data from the comparison of four groups (Table 3) demonstrated that the two detection results of two concentrations were identical before and after film application, and were 30% LEL and 40% LEL, respectively. There was a tiny difference in the trusted accuracy between the two detection results. However, the trusted accuracy can always reach 0.01 PPM. In the two concentration treatment groups, the modulus of four infrared signal detection channels were reduced by two-thirds after applying the black shading film.

### 4.4. High Humidity Environment Simulation Experiments and Results

**Figure 13:**
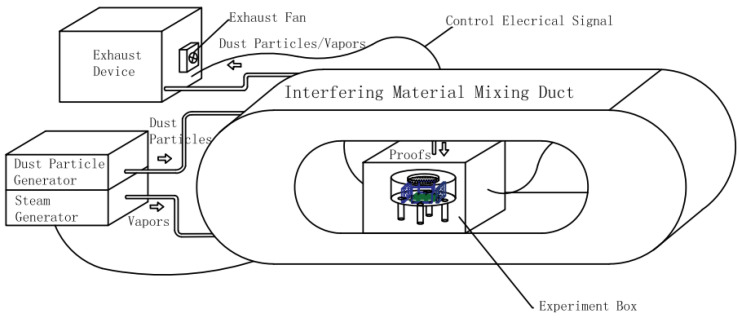
High humidity/high concentration of dust test device schematic.

We placed the sensor in the experiment box of the high-humidity/concentration dust test device (Figure 13) and set the humidity of the device at 85% to perform the high-humidity environment simulation experiment on the sensor. We opened the gas suction value to inject the methane gas with the concentrations of 20% LEL and 60% LEL into it when the humidity in the box had stabilized.

As shown in Table 4, the detection results of these two concentrations were 20% LEL and 60% LEL, and the trusted accuracy could still reach 0.012 PPM and 0.0061 PPM in a high-humidity environment of 85%. There was only a small reduction in the modulus of the four infrared signal detection channels.

### 4.5. High Concentration Dust Environment Simulation Experiments and Results

Similarly, we placed the sensor in the experiment box of the high-humidity/concentration dust test device and set the dust concentration to 100 mg/m^3^ in order that this dust concentration was used for the high-concentration dust environment simulation experiment. We injected the methane gas with the concentrations of 40% LEL and 80% LEL into it when the dust concentration in the box had settled down.

As shown in Table 5, the detection results of these two concentrations were 40% LEL and 80% LEL, and the trusted accuracy could still reach 0.0063 PPM and 0.0021 PPM in a high-concentration dust environment of 100 mg/m^3^. There was only a small reduction in the modulus of the four infrared signal detection channels.

### 4.6. High-Humidity and -Concentration Dust Environment Simulation Experiments and Results

Similarly, the sensor taped with designed shading film was placed in the experiment box of the high-humidity/concentration dust test device, and we set the humidity to 85% and the dust concentration to 100 mg/m^3^ for the high-humidity and -concentration dust environment simulation experiment. We injected the methane gas with the concentrations of 50% LEL and 90% LEL into it when the humidity and the dust concentration in the box had settled down.

As shown in Table 6, the detections of these two concentrations were 50% LEL and 90% LEL, and the trusted accuracy could still reach 0.029 PPM and 0.035 PPM in a high-humidity and -concentration dust environment. There was only a small reduction in the modulus of the four infrared signal detection channels.

## 5. Discussion

We compared the performance of the pyramid beam splitter methane gas concentration sensor with other mainstream sensors in the industry, as shown in Table 7. The other three sensors are evaluated in terms of % LEL level, which is significantly less accurate than the PPM level reached by the pyramid beam splitter sensor. The pyramid beam splitter sensor has a sensitivity level of PPM, which is significantly higher than the other three sensors. Only the pyramid beam splitter sensor has higher reliability in terms of redundancy and optical path design.

Compared with the present state of the advanced semiconductor material combustible gas sensors (such as metal oxide sensors, conducting polymer sensors, carbon nano-tube sensors, and 2D material sensors), the pyramid beam splitter methane gas concentration sensor provides higher sensitivity and accuracy and does not require special process treatment (such as that involving dopants or modifications), and it has greater environmental adaptability (especially in high-humidity and high-dust-concentration environments) and reliability (multi-channel redundant designs); additionally, it has a longer service life because it does not interact directly with the gas (unlike 2D material sensors) [22].

## 6. Conclusions

(1) The developed detector can effectively detect the methane gas from 0% LEL to 90% LEL, and the trusted accuracy of the detection result can reach 0.014 PPM. This illustrates that this detector can effectively detect the methane gas at each concentration with high accuracy through the new design of the sensitive element combined with the redundant four-channel methane gas concentration detection algorithm based on the CZT principle. Meanwhile, the method of multi-channel redundancy contributes to the improvement of the detector reliability to a certain degree.
(2) The design of the optical path structure of the sensitive element improves the sensor sensitivity so that it enables the effective detection of methane gas that is at less than the PPM level. The limit of the measurement concentration of this detector can reach 0.5 PPM, and the trusted accuracy is 0.01 PPM. The results indicate that the design of the optical path structure of the sensitive element improves the detector sensitivity so that it enables the effective detection of methane gas that is at less than the PPM level.
(3) The detector can be still operational, and the trusted accuracy of detection results can still reach 0.01 PPM under unfavorable conditions, with two-thirds of the option incident window of the sensitive element blocked, a humidity of 85%, and a dust concentration of 100 mg/m^3^. The results illustrate that the sensitive element based on the pyramidal beam splitter structure can improve detector reliability so that it can neutralize the effect of the optical window attached by contaminants.

## Figures and Tables

**Table 1:** Calculated methane gas concentration detection results for 0–90% LEL methane concentration.

Concentration (% LEL)	$M_{1}$	$M_{2}$	$R_{1}$	$R_{2}$	$C_{1}$	$C_{2}$	$C_{3}$	$C_{4}$	COL	$S_{COL}^{2}(PPM)$
0	1037.263935	1037.263932	1037.263937	1037.263936	0	0	0	0	0	-
10	933.537542	933.537539	1037.263937	1037.263936	0.1	0.1	0.1	0.1	0.1	0.0140
20	829.811148	829.811146	1037.263937	1037.263936	0.2	0.2	0.2	0.2	0.2	0.0120
30	726.084755	726.084752	1037.263937	1037.263936	0.3	0.3	0.3	0.3	0.3	0.0110
40	622.358361	622.358359	1037.263937	1037.263936	0.4	0.4	0.4	0.4	0.4	0.0090
50	518.631968	518.631966	1037.263937	1037.263936	0.5	0.5	0.5	0.5	0.5	0.0076
60	414.905574	414.905573	1037.263937	1037.263936	0.6	0.6	0.6	0.6	0.6	0.0061
70	311.179181	311.17918	1037.263937	1037.263936	0.7	0.7	0.7	0.7	0.7	0.0046
80	207.452787	207.452786	1037.263937	1037.263936	0.8	0.8	0.8	0.8	0.8	0.0030
90	103.726394	103.726393	1037.263937	1037.263936	0.9	0.9	0.9	0.9	0.9	0.0015

**Table 2:** Methane gas concentration detection limit test results.

$M_{1}$	$M_{2}$	$R_{1}$	$R_{2}$	$Q_{1}$	$Q_{2}$	$Q_{3}$	$Q_{4}$
1037.263401	1037.263451	1037.26395	1037.26394	0.999999471	0.999999481	0.999999519	0.999999529
$C_{1}$	$C_{2}$	$C_{3}$	$C_{4}$	COL	$S_{col}^{2}/PPM$	-	-
5.019 PPM	5.009 PPM	5.048 PPM	5.038 PPM	5.028 PPM	0.015	-	-

**Table 3:** Sensor anti-interference experimental calculation results.

Concentration (*% LEL*)	30 Pre-Film Data	30 Post-Film Data	70 Pre-Film Data	70 Post-Film Data
$M_{1}$	726.08	484.05	311.17	276.6
$M_{2}$	726.08	484.05	311.17	276.6
$R_{1}$	1037.2	691.5	1037.2	691.5
$R_{2}$	1037.2	691.5	1037.2	691.5
$C_{1}$	0.3	0.3	0.7	0.7
$C_{2}$	0.3	0.3	0.7	0.7
$C_{3}$	0.3	0.3	0.7	0.7
$C_{4}$	0.3	0.3	0.7	0.7
COL	0.3	0.3	0.7	0.7
$S_{COL}^{2}(PPM)$	0.0110	0.0186	0.0046	0.0071

**Table 4:** Experimental results of the sensor working in high-humidity environment.

Concentration (*% LEL*)	20	60
$M_{1}$	775.043612	387.52180
$M_{2}$	775.043610	387.52180
$R_{1}$	968.804517	968.80450
$R_{2}$	968.804516	968.80450
$Q_{1}$	0.80000	0.40000
$Q_{2}$	0.80000	0.40000
$Q_{3}$	0.80000	0.40000
$Q_{4}$	0.80000	0.40000
COL	0.20000	0.60000
$S_{COL}^{2}(PPM)$	0.012	0.0061

**Table 5:** Experimental results of the sensor working in high-concentration dust environment.

Concentration (*% LEL*)	40	80
$M_{1}$	458.0557537	150.4033
$M_{2}$	458.0557524	150.4033
$R_{1}$	763.4262576	752.0164
$R_{2}$	763.4262569	752.0164
$Q_{1}$	0.599999999	0.2000
$Q_{2}$	0.599999998	0.2000
$Q_{3}$	0.599999997	0.2000
$Q_{4}$	0.599999998	0.2000
COL	0.400000001	0.8000
$S_{COL}^{2}$ (PPM)	0.0063	0.0021

**Table 6:** Experimental results of the sensor working in high-humidity and high-concentration dust environment.

Concentration (*% LEL*)	50	90
$M_{1}$	311.1792	62.23584
$M_{2}$	311.1792	62.23584
$R_{1}$	622.3584	622.3584
$R_{2}$	622.3584	622.3584
$Q_{1}$	0.600000004	0.100000003
$Q_{2}$	0.600000003	0.100000006
$Q_{3}$	0.600000005	0.100000004
$Q_{4}$	0.600000004	0.100000003
COL	0.499999996	0.899999996
$S_{COL}^{2}$ (PPM)	0.029	0.035

**Table 7:** Comparison of sensor performance.

Model	Manufacturer	Accuracy	Sensitivity	Redundancy and Optical Path Reliability Design
PIR7000	Drager (German)	1% LEL	0.5% LEL	Not possessing
PIRECLB1	DET-TRONICS (UAS)	3–5% LEL	0.5% LEL	Not possessing
JTQB-BK61	BOKANG (China)	3–5% LEL	1% LEL	Not possessing
Pyramid beam splitter type sensor	HIT	0.5 PPM	0.01 PPM	Possessing

## Data Availability

The data are unavailable due to privacy or ethical restrictions.
